# Recent Advances on GaN-Based Micro-LEDs

**DOI:** 10.3390/mi14050991

**Published:** 2023-05-01

**Authors:** Youwei Zhang, Ruiqiang Xu, Qiushi Kang, Xiaoli Zhang, Zi-hui Zhang

**Affiliations:** 1 Guangdong Provincial Key Laboratory of Information Photonics Technology, School of Physics and Opto-Electronic Engineering, Guangdong University of Technology, Guangzhou 510006, China; 2 School of Integrated Circuits, Guangdong University of Technology, Guangzhou 510006, China

**Keywords:** GaN, micro-LED, non-radiative recombination, EQE, size effect

## Abstract

GaN-based micro-size light-emitting diodes (µLEDs) have a variety of attractive and distinctive advantages for display, visible-light communication (VLC), and other novel applications. The smaller size of LEDs affords them the benefits of enhanced current expansion, fewer self-heating effects, and higher current density bearing capacity. Low external quantum efficiency (EQE) resulting from non-radiative recombination and quantum confined stark effect (QCSE) is a serious barrier for application of µLEDs. In this work, the reasons for the poor EQE of µLEDs are reviewed, as are the optimization techniques for improving the EQE of µLEDs.

## 1. Introduction

GaN, as a direct band gap semiconductor material in the III group of nitrides, has a high radiation compound efficiency and is ideal for the fabrication of light-emitting devices. Modern society has entered into information technology and is moving towards intelligence, with display and communication being the key links to information exchange and intelligence. With the accelerated development of wireless communication and big data technologies in recent years, the demand for data transmission rates and communication capacity is increasing day by day. Based on human development needs, GaN-based micro-size light-emitting diodes (µLEDs) have received significant research attention in the fields of display and visible light communication (VLC).

µLEDs-based display technology is a self-illuminating display technology that produces an image by combining a matrix of micron-sized LED light-emitting devices on an actively addressable driver substrate for individual control and illumination [1]. µLED displays offer several benefits, including self-illumination, high efficiency, excellent integration, low power usage, and considerable stability; they are also tiny, flexible, and simple to disassemble and combine, and they can be utilized in any existing display application, ranging from small to large size [2]. When compared with organic light-emitting diodes (OLEDs) and liquid crystal displays (LCDs), µLEDs have the benefit of having superb image quality in addition to superior stability and other remarkable qualities. Compared to LCDs, because self-emitting µLEDs do not require color filters or backlight modules, the thickness of the device can be decreased. In addition, the brightness of µLEDs can reach up to 100,000 cd/m^2^, and a µLEDs-based display has a response time of 0.2 ns [3], which is ~10^4^ times faster than in an OLED. Besides, the Pixels Per Inch (PPI) of µLEDs displays can exceed 1500 [4]. The Jade Bird Display (JBD) has demonstrated a high-pixel density display with 10 000 PPI. According to the Arrhenius formula, µLEDs are expected to have a lifetime that is higher than 10 years, which places them in a more competitive position than LCDs and OLEDs. Besides, µLEDs have a great color gamut and a broad view angle in addition to their other advantageous characteristics [5,6,7]. More details on the performance comparison between them can be found in Table 1. µLEDs integrate the majority of the benefits that are offered by LCDs and OLEDs, and µLEDs have the potential to save more energy since they provide greater light power.

However, µLEDs display technologies still face obstacles such as full-color display and massive transfer. Currently, manufacturing red, green, and blue (RGB) micro-LED pixels on the same wafer by local strain engineering and growth, exciting quantum dots (QDs) for full color, and transfer printing RGB microLEDs are all viable routes to producing full-color displays [9,10,11]. Blue/ultraviolet (UV) flip-chip or vertical chip microLEDs may also be used to convert colors to make RGB microLEDs. Using microLEDs to excite fluorescent materials for color conversion, one can simply make monochromatic microLEDs and change their colors. Typically, blue/UV LEDs are used to excite red and green quantum dots or phosphors, respectively, to produce red and green light [12,13,14]. Due to their huge particle sizes of several micrometers [15,16] and their size-related brightness uniformity [12,13], the application of phosphors is limited. As a result, the color conversion efficiency of microLEDs after phosphor coating is relatively low [17]. It is possible to remedy this issue by reducing the size of the phosphor, but doing so might reduce its quantum efficiency [18,19]. More specifically, UV microLEDs are better suited than blue microLEDs for stimulating color conversion materials. This may result in an uneven distribution of the luminous intensity of differently colored microLEDs as well as inconsistent responses from microLEDs since the excitation efficiency of blue light is relatively low and its response time is quicker than that of red and green light [10]. A key method for achieving single-chip microLED displays is color conversion technology. However, a significant issue influencing the longevity and dependability of full-color microLED displays is the stability of quantum dots. In addition, heavy metals that are detrimental to human health might be present in quantum dots, and the spatial separation between MQW and quantum dots limits the efficiency of color conversion [20]. There is still a holdup in the massive transfer of µLED. The necessity of a very stable and accurate transfer process is one of the primary technological hurdles. There have been quite a few review articles on how to make µLEDs-based displays [21,22,23]. In addition to display applications, the use of miniature LED chips can be effective in increasing system modulation bandwidth and response speed in visible light communication applications [24,25,26,27,28].

µLED-based visible light communication technology uses light in the visible wavelength band as the source of data signals, which are transmitted by driving LEDs to blink rapidly to achieve the purpose of transmitting data [29,30,31,32,33]. Due to the reduced chip size, this can directly reduce the device capacitance and thus the resistive-capacitive (RC) time delay. In addition, the smaller active area facilitates the spreading and uniform distribution of the current, which can significantly increase the maximum tolerable operating current density of the chip and thus shorten the carrier lifetime [34,35,36], thus expanding the modulation bandwidth of the LED chip. When compared to traditional free-space optical communication methods (which often make use of the infrared band), the utilization of visible µLEDs as emitters does not necessitate the application of special power restrictions. Not only does it help ease the rising shortage of spectrum, but it also guarantees the safety of data and makes it easier to integrate systems. Additionally, VLC offers a number of other advantages: no electromagnetic radiation, no pollution, no harm to humans, and other significant advantages.

Typically, the working current density is high in VLC sources so that a greater communication range, improved signal-to-noise ratio (SNR), and expanded bandwidth can be attained. Under the condition of high current density, the size-dependent effect and the droop effect cause a reduction in the efficiency of the device. Therefore, there is a contradiction between the bandwidth and efficiency of µLEDs. It is of the utmost importance that the droop effect be optimized. In addition, there have been quite a few review articles on high-speed visible light communication based on microLEDs [37,38].

**Figure 1:**
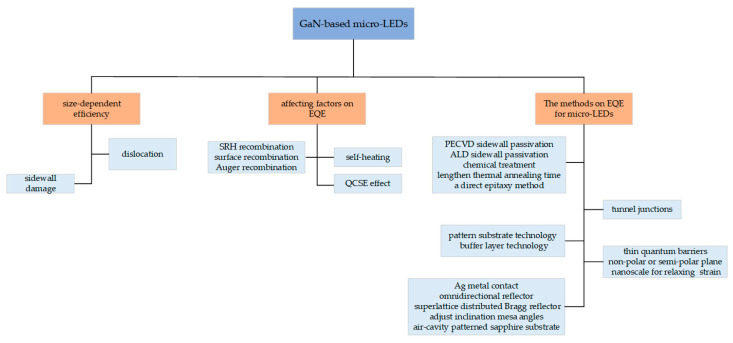
Structure diagram of this paper.

In this review, we introduce the size effect of µLEDs and discuss the factors that influence the EQE of µLEDs. In addition, we review various approaches that have been taken in recent years to enhance the EQE of µLEDs. More details can be found in Figure 1. The information covered in this work is crucial to comprehending the physics of the µLED device, and the methods summarized are useful to motivate the community to make µLEDs with better performance.

## 2. Size-Dependent Efficiency

The decrease in efficiency related to size is a key challenge affecting µLED applications, and the development of the smallest possible device size is affected by efficiency limitations. Here, we mainly focus on discussing the two most crucial elements: sidewall damage and dislocation density. Understanding the impact and causes of these two components can provide assistance in analyzing and improving device performance.

### 2.1. Side-Wall Damage

Inductively Coupled Plasma (ICP) can cause etching damage near the chip sidewall during the manufacturing process of µLED devices. As the size of LED devices gradually decreases, the proportion of sidewalls to the total area of the device gradually increases, and the proportion of defects formed by etching damage gradually increases. These defects lead to a gradual increase in the proportion of non-radiative recombination, reducing the luminous efficiency. At the same time, new leakage channels are introduced to accentuate the reverse leakage of the device [39,40]. Surface recombination is another key factor in the efficiency performance related to size in µLEDs. Surface recombination is the behavior wherein the concentration of charge carriers significantly decreases near the surface. Smaller devices are more susceptible to the detrimental effects of sidewall damage and surface recombination, which reduce their efficiency. Sidewall defects and surface recombination have been demonstrated to decrease carrier injection efficiency [41], notably in p-type domains.

### 2.2. The Existence of Dislocations

Sapphire is the most commonly used substrate for GaN-based blue and green LEDs. The disadvantage is that it has a large lattice mismatch and thermal expansion mismatch with GaN. The large lattice mismatch leads to a high dislocation density in the GaN epitaxial layer, and the dislocations reduce carrier mobility and minority carrier lifetime, which reduces thermal conductivity. The thermal mismatch creates stress during the cooling of the epitaxial layer, which can lead to cracking and ultimately degrade device performance.

The dislocation from LED to mini-LED (>100 µm) has little effect on the performance of the chip. This is because the migration distance of minority carriers in GaN is very small, and so the effect of dislocation on the luminescence efficiency of GaN-based materials is very small [42]. However, the adverse effect of dislocations on the performance of GaN-based µLED chips (<100 µm) increases with decrease in chip size. Dislocations are also non-radiative recombination centers in GaN [43], and as small leakage channels, dislocations have a significant impact on the voltammetry of p-n junctions under low current conditions. A µLED has a small size and requires a low injection current density, making its requirements for dislocation density more stringent. The distribution of dislocations in the GaN epitaxial layer is uneven. When the chip size is small to a certain extent, even the difference in the number of dislocations between adjacent chips in the same epitaxial layer will be very large. Therefore, it is necessary to enhance the crystal quality to improve the consistency of chip performance.

## 3. Affecting Factors on the EQE of µLEDs

The low external quantum efficiency (EQE) of µLEDs is one of the difficulties in making efficient displays and enhancing the capabilities of communication systems. It is therefore crucial to enhance the EQE of µLEDs, which is influenced by a combination of internal quantum efficiency (IQE) and light extraction efficiency (LEE), as expressed in Equation (1):

(1) EQE=IQE×LEE

EQE is an important indicator of LED performance, defined as the ratio of photons emitted from the device to the number of carriers injected into the device. IQE is the ratio of radiative compounding divided by the ratio of total compounding (both radiative and non-radiative), and LEE represents the photons emitted from outside the device through the photons generated by compounding. The IQE can be studied using the traditional ABC approximation model [44].

(2) $$IQE=\frac{{\mathrm{Bn}}^{2}}{An+Bn^{2}+Cn^{3}}$$

In this model, the coefficients *A*, *B*, and *C* are related to the Shockley-Read-Hall (SRH) recombination, radiation recombination, and Auger recombination, respectively. *n* is the quantum well carrier concentration.

### 3.1. Non-Radiative Recombination Effect for µLEDs

**Figure 2:**
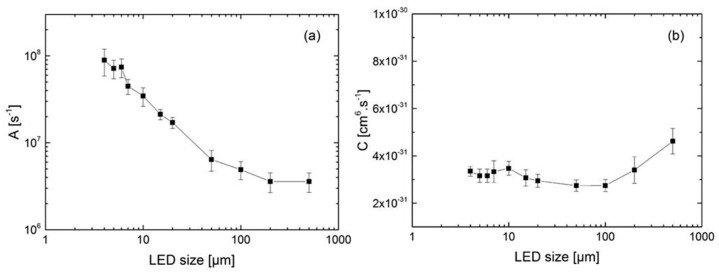
Plot of the extracted coefficients A (**a**) and C (**b**) vs. LED size. Reproduced from Ref. [47], with the permission of AIP Publishing.

µLEDs are LEDs with dimensions less than 100 µm. Due to their small size, µLEDs have been found to have a diminishing maximum EQE as device dimensions get smaller [45,46]. Olivier et al. showed that the Shockley-Read-HaII (SRH) recombination rate increases when the µLED size is reduced [47], as shown in Figure 2a. However, the Auger recombination coefficient is almost unaffected by µLED size and remains constant, as shown in Figure 2b. The plasma dry etching technique is by far the most widely used method for defining the light-emitting mesa. Due to the large perimeter/area ratio (P/A) of µLEDs compared to large conventional LEDs, the effect of etching processes causing high-density surface defects on the sidewalls becomes more pronounced as the chip size decreases [46].

**Figure 3:**
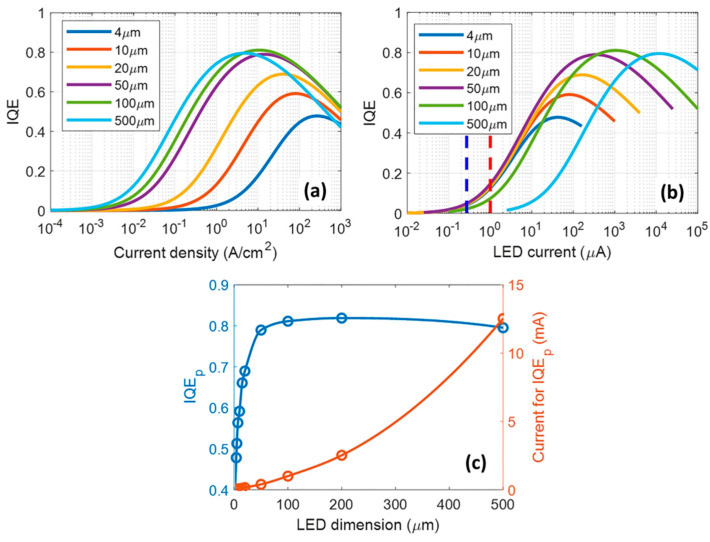
(**a**) The relationship between IQE and current density and (**b**) current for LEDs of varying sizes. (**c**) Related current levels and peak IQEs for various LED sizes. The biue and red lines are fits to the experimental data (blue and red circles). Reproduced from Ref. [50], with the permission of John Wiley and Sons.

SRH non-radiative recombination is facilitated by surface defects and dangling bonds on the surfaces of crystals, which act as traps in the bandgap. Since µLEDs have a better current-spreading effect [48,49], carriers will reach the sidewalls more easily, surface compounding will be more significant, and performance losses will be more severe. It is worth noting that the carrier trapping effect will be weakened by sidewall defects as a result of the shorter carrier lifespan at high currents, which will lead to peak efficiencies at larger current densities. The peak efficiency decreases significantly with decreasing chip size, and the increase in efficiency flip current density is also an indication of enhanced surface non-radiative compounding [50]. As shown in Figure 3, the blue dashed line indicates that µLED is typically driven at very low current levels, and the red dashed line indicates that higher current levels of 1 µA are necessary to generate an adequate amount of display brightness. The peak EQE and rollover current are shown for various sizes of LED chips in Figure 3c. This figure shows that when the size of the LED chip is reduced to below 50 × 50 µm^2^, the peak EQE drops considerably.

**Figure 4:**
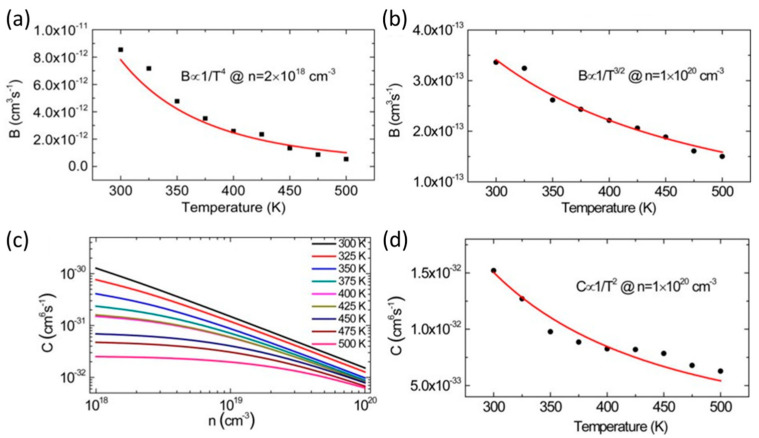
Temperature-dependent coefficient B alterations (**a**) at *n* = 2 × 10^18^ cm^−3^ and (**b**) at *n* = 1 × 10^20^ cm^−3^. The red lines are fits to the experimental data (black symbols) in (**a**,**b**). (**c**) Calculated coefficient C in relation to n at various temperatures. (**d**) Alterations in coefficient C with temperature at *n* = 1 × 10^20^ cm^−3^. The red line is a fit to the calculated data (black symbols) in (**d**). Reproduced from Ref. [52], with the permission of AIP Publishing.

Moreover, the current injection for LEDs with tiny chips must be low in order to prevent efficiency drooping when the levels of injection current density are high. These locations have more details on the correlation between current density and EQE [49,51]. Tian et al. reported a trend in the correlation of the complex coefficient with respect to temperature for different injected carrier concentrations [52]. The temperature changes of the B coefficient at *n* = 2 × 10^18^ cm^−3^ and *n* = 1 × 10^20^ cm^−3^ are shown in Figure 4a,b, respectively. At low carrier concentrations, increasing temperature results in a considerable reduction in the B coefficient. However, when carrier concentrations are high, the B coefficient becomes less affected by temperature. As the temperature rises, the coefficients B and C decrease. Moreover, at higher carrier concentrations, the dependence of coefficients B and C on temperature is reduced, as shown in Figure 4b,d. Figure 4c investigates the trend of the coefficient C with temperature for different carrier concentrations from 300 K to 500 K. The coefficient C falls as temperature rises for all carrier concentrations. A decreased temperature dependency is acquired when the carrier concentration rises, just like in the case of coefficient B. It is important to note that this report’s temperature dependence of the coefficient C defies the majority of theoretical and experimental trends in the literature [53,54,55,56], but it is consistent with the theoretical outcome of one design that has weak carrier confinement in the quantum wells (QWs) [57].

### 3.2. Thermal Effect for µLEDs

Sapphire has a very low thermal conductivity and does not dissipate heat easily compared to other substrate materials. This makes the thermal management of µLEDs important. The benefits of silicon substrates are low cost, vast area, high quality, and excellent conductivity and heat conductivity. Silicon has a thermal conductivity that is five times greater than sapphire, and silicon substrate LEDs have high performance and a long lifetime due to their efficient heat dissipation. However, GaN and silicon have greater lattice mismatches and thermal stress mismatches, which result in more defects in the epitaxial layer during growth [58,59,60]. Currently, epitaxial growth of LEDs on sapphire substrates is the most marketable and lowest-cost technology. We all know that when an LED is in operation, it generates a large amount of joule heat, which may cause the EQE to flip [61]. Fortunately, related research has demonstrated that the self-heating effect is chip-size dependent, with smaller pixels having a slower increase in junction temperature [24,62]. The thermal resistance has been reported to decrease linearly with the size of µLEDs [63]. In addition, improved LEE can result from device size decreases [64], and improved LEE effectively weakens free carrier absorption, but crosstalk in display applications may result from the majority of photons escaping from the sidewall [65]. Therefore, research efforts should also be made to better manage photon propagation.

### 3.3. QCSE for III-Nitride LEDs

The quantum confined Stark effect (QCSE), which causes the emission wavelength to shift with increased injection current, is a long-standing issue with group III-nitride LEDs. The effect is caused by the intrinsic polarization field in the InGaN/GaN multiple quantum wells (MQWs) region. Spontaneous polarization and piezoelectric polarization make up the polarization of group III nitrides. The absence of inversion symmetry in a specific crystal orientation is what causes spontaneous polarization. Stresses produced by strained films, including in the InGaN/GaN, are what cause piezoelectric polarization [66].

Another factor that is QCSE contributing to the low EQE of LEDs is the tilting of the energy band structure caused by the polarizing electric field, which has a significant impact on the overlap of the electron-hole wave function and lowers the radiative combination rate of the carriers. InGaN/GaN MQW active regions obtained on the c-plane are the foundation of the most mature and conventional LEDs. GaN-based LEDs, especially those with a high In component, are severely affected by QCSE. McKendry et al. investigated the performance of LEDs with different pixel diameters and wavelengths [35]. Devices at different wavelengths exhibit significantly different maximum modulation bandwidths under a uniform growth process. In addition to device homogeneity issues, this may be due to the fact that the coefficient ABC alters with In content [67]. The coefficient B may not vary considerably when the content of In increases, whereas the coefficient C may rise by 50%. The results of the study indicate that shorter carrier lifetimes are caused by much greater non-radiative combinations at high current, while In-rich clusters at high current densities have carriers that spill out of the clusters and participate in surrounding SRH complexes. When the carrier density is high, the carriers may help shield the QCSE, which is a significant justification for enhancing LED modulation performance at high current densities [68], but this is unreasonable since efficiency degradation and heat dissipation can be severe issues. Therefore, a number of countermeasures should be taken to mitigate QCSE.

## 4. Solution for Increasing EQE of µLEDs

Many efforts have been made to improve the EQE of µLEDs. On the one hand, the IQE has been improved by reducing non-radiative recombination and enhancing radiative recombination, and on the other hand, the device structure has been optimized to improve the LEE. There are several approaches for increasing the EQE of µLEDs.

### 4.1. Defect Density Control

**Figure 5:**
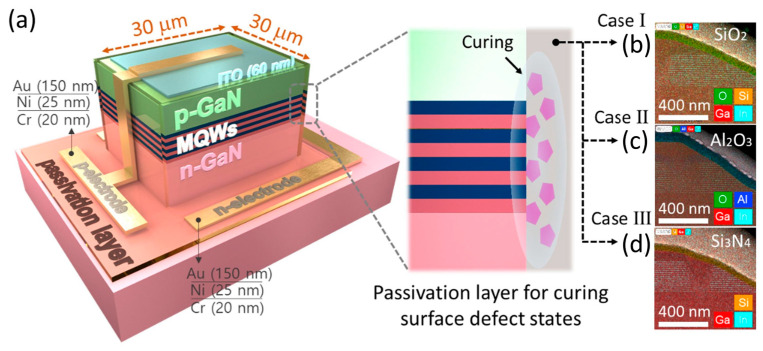
(**a**) Device structure for µLEDs (left) and effect of surface passivation for various passivation layers (right). µLEDs in high-angle annular dark field scanning transmission electron microscope (HAADF-STEM) images with (**b**) SiO_2_, (**c**) Al_2_O_3_, and (**d**) Si_3_N_4_ passivation layers. Reproduced from Ref. [69], with the permission of Elsevier.

By reducing the density of sidewall flaws brought on by mesa etching, IQE is enhanced. In order to ascertain the potential mechanism of passivation, Kyung et al. used three passivation materials, SiO_2_, Al_2_O_3_, and Si_3_N_4_, to investigate the chemical bonding properties at the sidewall/passivation layer interface [69]. The device structure is shown in Figure 5. They found that SiO_2_ passivation was more effective than Al_2_O_3_ and Si_3_N_4_ passivation in reducing sidewall defects, and µLEDs with SiO_2_ passivation exhibited high photoluminescence (PL) efficiency, high optical output power, and high current density due to the fact that the Ga-O bond formation energy was lower than the Si-O bond dissociation energy; at the interface between the GaN and the passivation layer, a great deal of Ga-O bonds were formed.

**Figure 6:**
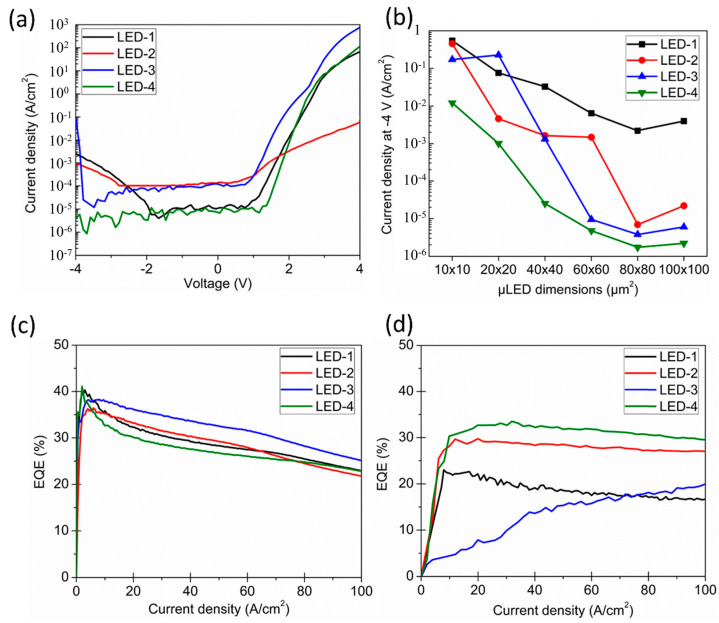
(**a**) Properties for current density-voltage of 20 × 20 um^2^ µLEDs with various sidewall passivation approaches. (**b**) The relationship between the sizes of LEDs with various sidewall passivation techniques and leakage current density at −4 V. The reliance of EQE on current injection for devices with various sidewall passivation techniques (**c**) 100 × 100 µm^2^ and (**d**) 20 × 20 um^2^. Reproduced from Ref. [73], with the permission of Optical Society of America.

Passivation layers are deposited on µLEDs by plasma-enhanced chemical vapor deposition (PECVD) and atomic layer deposition (ALD). The PECVD system provides fast deposition rates. According to the results of experiments, the electrical and optical characteristics of the devices can be somewhat improved by the passivation layers grown by the PECVD systems, for example, by improving light extraction efficiency and reducing leakage currents [70,71]. However, because PECVD methods are unable to produce passivation layers with extremely compact atom arrays, some surface defects may not be effectively passivated [72]. Therefore, due to the extremely compact atomic arrays and the accurately controlled atomic-level layer deposition technology, the ALD system is another, more satisfactory solution. PECVD sidewall passivation was shown to be superior in lowering the leakage current of big devices in a study by Wong et al. on the performance enhancement of µLEDs utilizing ALD systems [73], but it was ineffective in reducing leakage current of µLEDs smaller than 60 × 60 µm^2^. As shown in Figure 6a, LED-1 is a µLED without sidewall passivation, LED-2 is a µLED with inductively coupled plasma (ICP) etching and sidewall passivation applied using the ALD process, LED-3 is a µLED with hydrogen fluoride (HF) etching and sidewall passivation applied using the PECVD process, and LED-4 is a µLED with HF etching and sidewall passivation applied using the ALD process. Leakage current densities in LED-2 and LED-3 are an order of magnitude greater between −2 V and 0 V than in LED-1 and LED-4 when the device size is 20 × 20 µm^2^. LED-2 and LED-3 with high leakage current densities indicate that ICP etching and PECVD create more leakage channels in the µLEDs. As shown in Figure 6b, PECVD sidewall passivation produces significantly worse results in µLEDs smaller than 60 × 60 µm^2^ compared to ALD. Figure 6c, when compared to Figure 6d, shows that PECVD sidewall passivation produces better results in larger-sized LEDs due to the relatively small perimeter/area and the lack of impact of sidewall damage on the performance of larger-sized devices. Figure 6d reveals that for 20 × 20 µm^2^ µLEDs, the EQE of LED-4 based on ALD passivation and HF etching is higher than that of LED-3 based on HF etching and PECVD passivation, while the comparison of LED-1 and LED-2 further highlights the significance of the ALD technology for sidewall passivation.

**Figure 7:**
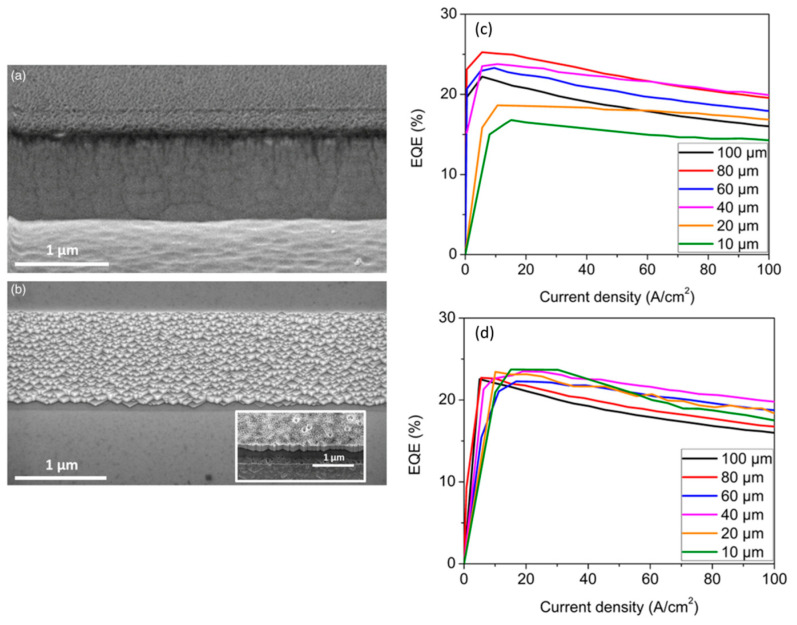
Images taken using scanning electron microscopy to compare the sidewall profile of µLEDs (**a**) before and (**b**) after KOH chemical treatment. Size-dependent properties of EQE in relation to current density for μLEDs (**c**) without and (**d**) with sidewall treatment. Reproduced from Ref. [78], with the permission of The Japan Society of Applied Physics.

Alternatively, chemical treatment is an effective method of reducing the defect density of sidewall treatments, and device performance is expected to be further enhanced by the incorporation of chemical treatment and ALD sidewall passivation. The fundamental theory of chemical processing is to passivate or oxygenate the semiconductor using wet chemicals in order to create a larger band gap material at the surface interface and produce a surface with reduced sidewall damage, surface compounding, and dangling bonds. Plasma damage can be eliminated by chemical treatments employing hydrochloric acid, ammonium sulfide, and potassium hydroxide (KOH), according to reports [74,75,76,77]. The leakage current has been shown to be reduced by chemical treatment. According to Wong et al. [78], a sidewall passivation study incorporating ALD and chemical treatment was conducted. Scanning electron microscopy (SEM) images of the LED sidewall profile before and after the KOH chemical treatment are shown in Figure 7a,b, respectively. The etched nature produced a rough sidewall surface for the non-KOH treated devices. The KOH-treated sidewall developed m-plane faceted features with dimensions ranging from 50 to 200 nm. Previous investigations utilizing tetramethylammonium hydroxide (TMAH) or KOH have revealed the creation of m-plane facets on the device walls [79,80]. Figure 7c,d show the EQEs regarding current density injection in the absence and in combination with KOH and ALD sidewall treatments, respectively, with the EQEs of the larger device sizes being almost unaffected by KOH and ALD sidewall treatments. The peak EQE of the 100 × 100 µm^2^ µLED appears at the current density of 5 A cm^−2^, while the peak EQE is displayed at 15 A cm^−2^ when the device size is reduced to 10 × 10 µm^2^. Besides, although the efficiency of these devices is improved by the sidewall treatment, the effect of non-radiative compounding is not completely eliminated, which is reflected in the reduced peak efficiency of the small devices.

In addition, by lengthening the thermal annealing time, sidewall defects can be repaired in part, enhancing efficiency at low injection current densities [46,76]. The quantum efficiency of small-size LEDs is considerably lower than that of large-size LEDs as a result of the dry etching process, which becomes a considerable problem when the LED size is ≤10 µm. A direct epitaxy method for µLEDs in which the dry etching technique used to form the µLED mesa is no longer required was conceived and developed by the Sheffield team and is called the “confined selective epitaxy” (CSE) approach [81]. Steady emission color, extremely high external quantum efficiency, reduced leakage current, improved indium integration to generate red emissions, and low parasitic capacitance are its many main benefits. This approach could be a candidate for the manufacture of high-quality µLEDs for micro-size displays and VLC applications.

### 4.2. Managing the Spread of Current

**Figure 8:**
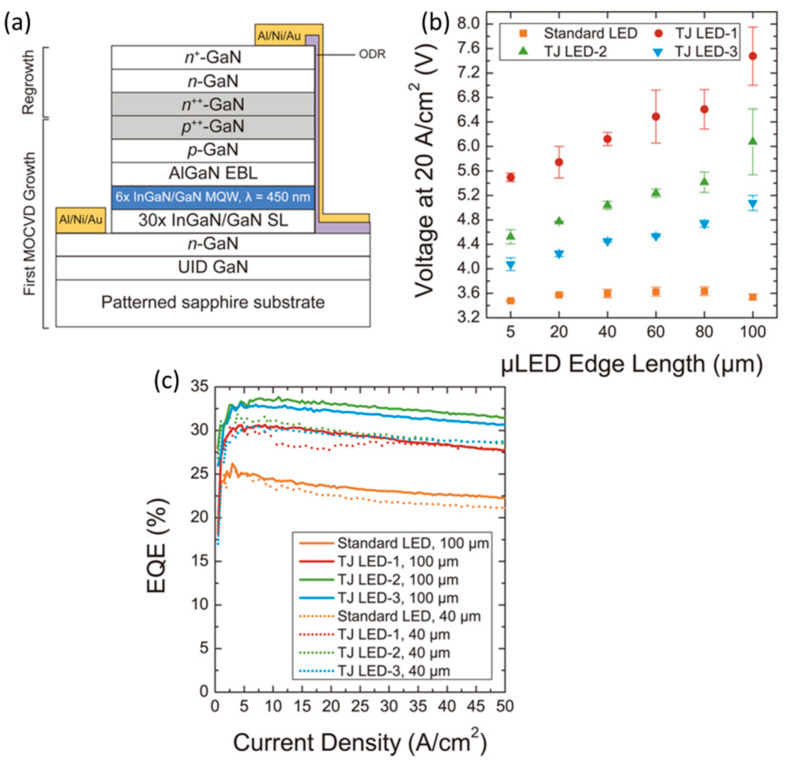
(**a**) Cross-section diagram of the treated µLEDs and layers produced using MOCVD. (**b**) Voltage for various µLEDs with the 20 A/cm^2^ injection current density. (**c**) EQE in relation to current density for different µLEDs with sizes of 100 × 100 µm^2^ and 40 × 40 µm^2^. Reproduced from Ref. [88], with the permission of The Japan Society of Applied Physics.

Using buried tunneling junctions (TJ) is another effective way to improve devices [82]. Metal organic chemical vapor deposition (MOCVD) and molecular beam epitaxy (MBE), two regenerative growth processes that both have advantages and disadvantages for device performance, have been used to achieve tunnel junctions. When tunnel junctions are created via MBE, the hydrogen level stays low while they are growing, and the conductivity of the p-GaN layer stays constant after they have grown. However, the utilization of MBE-formed tunnel junctions for large-scale manufacturing is severely hampered because of scalability [83,84,85]. The resistivity of p-GaN dramatically rises with hydrogen passivation at high hydrogen concentrations during progression, although MOCVD-formed tunnel junctions are another option due to their great reactor scalability [86,87]. TJ allows better control of the current injected into the µLED diode active layer, thereby reducing carrier arrival at the table edge, which effectively suppresses surface non-radiative recombination. This approach was applied to the tunneling connection method for GaN-based µLEDs [88]. Figure 8a shows the structure of a µLED with TJ contacts grown by MOCVD. The standard LEDs in Figure 8b,c represent LEDs without TJ structures with an epitaxial layer of indium tin oxide (ITO), while LED-1, LED-2, and LED-3 have TJ structures that replace the ITO layer and have different silicon concentrations. We can observe that the poor efficiency of p-GaN doping causes the forward voltage of the LED to increase [89], which makes the TJ structure less probable for carrier tunneling from the valence band of p^++^-GaN to the conduction band of n^++^-GaN under reverse bias. Figure 8c shows that the three TJ µLEDs have a higher peak EQE, which can be due to more uniform current distribution on the p-side and n-side, and that n-GaN is more optically transparent than ITO compared to a standard LED, which enhances the light extraction efficiency.

### 4.3. Mitigate the QCSE Effect

**Figure 9:**
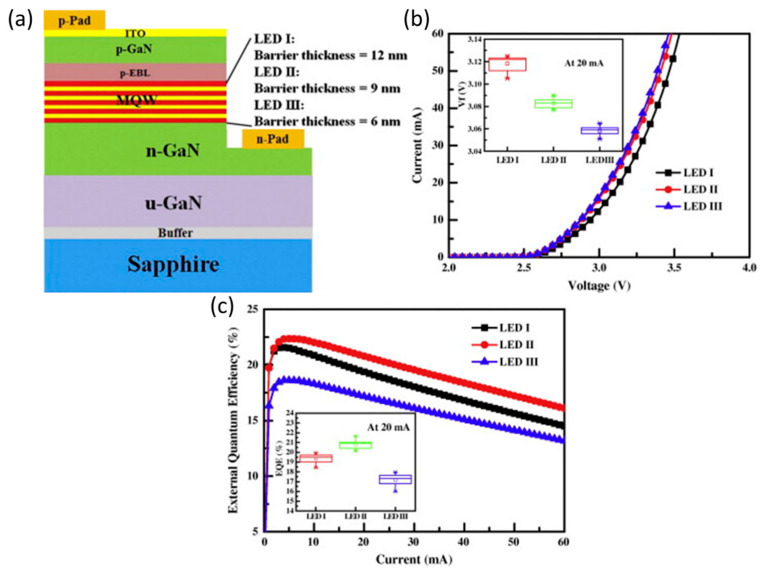
(**a**) The structure diagrams of different QB-thickness LEDs. (**b**) The current–voltage properties for three fabricated LEDs. (**c**) The EQE as a function of injection currents for various LEDs. Reproduced from Ref. [91], with the permission of Elsevier.

As mentioned above, GaN-based LEDs, particularly In-rich red and green µLEDs, suffer greatly from the extremely strong QCSE present in InGaN/GaN-based MQWs. This seriously worsens the IQE of GaN-based µLEDs. The QCSE has been reported to be mitigated by the optimization of epitaxial structures. For example, thin quantum barriers (QBs) in MQWs are crucial for reducing carrier lifetimes [90,91,92], which can decrease the polarization field inside the QW, enhance hole injection, and result in greater uniformity in carrier distribution across the active region. The schematic architectures of the LED I, LED II, and LED III samples are shown in Figure 9a, and their corresponding GaN QB thicknesses are 12, 9, and 6 nm. Figure 9b demonstrates the forward current-voltage properties of three manufactured LEDs. As the thickness of the QB shrinks, the forward voltage reduces. This may be explained by the fact that series resistance decreases as the thickness of QBs decreases. The series resistances for LEDs I, II, and III were measured to be 7.79, 7.28, and 6.93 Ω, respectively. The EQE of three manufactured LEDs is depicted in Figure 9c as a function of forward current. It is evident that LED II, which had a 9 nm QB thickness, had the highest performance. At an injection current of 60 mA, the efficiencies of LEDs I, II, and III are decreased by 32.7%, 27.9%, and 29.2%, respectively, from their maximum efficiencies. It has further been shown that the luminous intensity of the samples decreases significantly as the thickness is further reduced to 5 nm. This may be caused by quantum tunneling-assisted leakage of carriers and deterioration in crystal quality, which lead to increased non-radiative losses [93]. Therefore, designing extremely thin QB samples requires fine interface quality control to meet theoretical expectations. A report on the improved performance of µLEDs by reducing QB thickness can be found here [94], and the improved EQE of µLEDs is obtained from both numerical calculations and experimental results.

**Figure 10:**
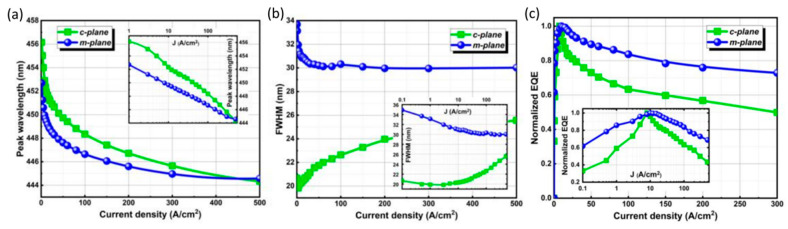
The comparisons of c-plane and m-plane µLEDs. (**a**) Peak wavelength, (**b**) full width at half maximum (FWHM), and (**c**) efficiency attenuation as a function of current density. Reproduced from Ref. [98], with the permission of John Wiley and Sons.

By altering the growth plane to a non-polar or semi-polar plane, the impact of polarization can be diminished or perhaps completely eliminated, offering a fundamental solution to this issue [26,95,96,97,98]. Stabilization of wavelengths has been demonstrated by MOCVD epitaxial growth of multiple quantum wells on non-polar or semi-polar planes [98], as shown in Figure 10a. Peak wavelength shifts for the m-plane and c-plane LED devices are 8.12 nm and 11.81 nm, respectively. Figure 10b shows that the m-plane devices have a slower decreasing trend, which indicates some stability, and do not shift any longer under high current density injection. This may be attributed to the decrease in the m-plane polarization field, which improves the energy band tilt phenomenon. However, it can be attributed to high-density stacking mistakes in the epitaxy method that the c-plane device has a considerably narrower full wavelength at half maximum than the m-plane device. Figure 10c illustrates that as the current density increases, the efficiency of c-plane decays severely compared to the m-plane and that the EQE of the m-plane is higher than that of the c-plane at high current densities. However, high-quality non-polar and semi-polar GaN-based µLEDs are grown on substrates with special crystal orientations due to cost and crystal quality issues. They are therefore not suitable for large-scale production. µLEDs are currently more suitable for growth on [0001] oriented Si [99,100] and sapphire substrates [72], as they are easier to fabricate. In addition, by progressively decreasing the dimension from the micron scale to the nanoscale, strain can be released [101,102]. The fabrication of nano-LEDs, such as nanoring, nanorod, anti-nanoring, and nanohole LEDs, using a new colloidal photolithography technique based on the Talbot effect has been reported. These LEDs have lifetimes of 4, 6, 8, and 12 ns in the typical time-resolved photoluminescence test, as opposed to 15 ns for big-area LEDs [103]. However, the manufacturing process for nano-LEDs is more complex and requires special lithography systems and processes.

### 4.4. Improving the Crystalline Quality

**Figure 11:**
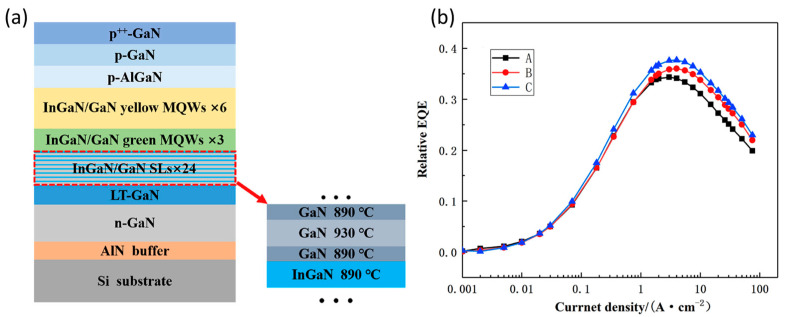
(**a**) Schematic epitaxial structure with preparation layer. (**b**) Room-temperature EQE as a function of current density. Reproduced from Ref. [105], with the permission of Elsevier.

Pattern substrate technology, buffer layer technology, and other technologies are more widely used in the heteroepitaxial growth of GaN on sapphire or silicon substrates to reduce dislocation density and improve crystal quality [39]. The homogeneous epitaxial technology of high-quality GaN substrates can obtain high-quality LED epitaxial wafers, but this substrate is costly, and it is tough to obtain large-sized epitaxial wafers. Buffer insertion layers such as GaN and AlN are often used in the epitaxial growth of sapphire-based LEDs [104], and the buffer layers in the preparation of silicon substrate LEDs usually include AlGaN/GaN, AlN/GaN superlattice and so on [105,106]. Nucleation centers can be provided by the insertion of buffer layers for GaN growth, and this can reduce the dislocation density of GaN. Yang et al. filtered dislocations by growing a single-layer AlN buffer layer and an optimized superlattice insertion layer on a silicon substrate [105]. Figure 11a,b, respectively, show the epitaxial structure and EQE of the device as a function of current density. At the current density of 4 A cm^−2^, the green and the yellow LED EQE with a luminescent wavelength of 551 nm are increased to 37.7%. Chen et al. obtained nano-patterned sapphire substrate (NPSS) by nanoimprinting and growing GaN on NPSS substrate [107]. The stress in the GaN thin film could be effectively relaxed. The dislocation density decreases to 1.8 × 10^8^ cm^−2^, and surface roughness was reduced to below 0.1 nm.

### 4.5. Increasing the LEE for µLEDs

**Figure 12:**
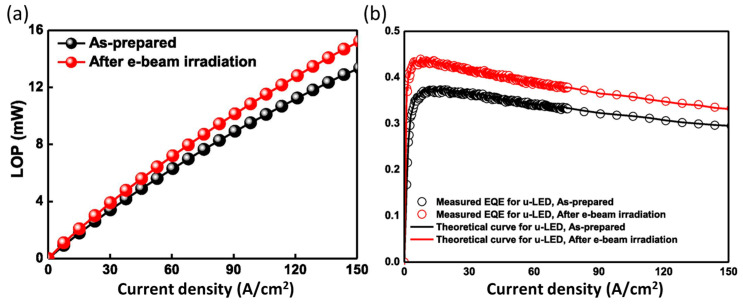
The comparisons with and without EBI on Ag thin film-based µLEDs. (**a**) Light output power (LOP) properties, (**b**) EQE properties. Reproduced from Ref. [108], with the permission of Elsevier.

For conventional flip-chip µLEDs, a highly reflective Ag metal contact fabricated by using electron beam irradiation (EBI) is proposed for better light extraction from the substrate [108]. Figure 12 demonstrates that the light output power (LOP) of the EBI-based µLED is greater than that of the unbased device. Moreover, at the current density of 83 A/cm^2^, EBI-based µLED has a greater EQE (37.2%). As can be seen in Table 2, the IQE at drive current of the two types of LEDs is almost equal, and the rise in EQE is caused by a rise in LEE.

Due to the increase in critical angle, sidewall passivation can improve light extraction [70,109]. Hwang et al. deposited an omnidirectional reflector (ODR) structure on the sidewall of a µLED by using ion beam deposition [45], which can reduce light loss and enhance the light extraction efficiency for µLEDs. ODR structures are mainly composed of silicon dioxide (SiO_2_) and silicon pentoxide (Ta_2_O_5_). SiO_2_ and Ta_2_O_5_ are alternately overlapped, with Ta_2_O_5_ being deposited in the outermost layer. The carefully designed reflective structure reflects the light into the µLED, ultimately improving the performance of the device.

**Figure 13:**
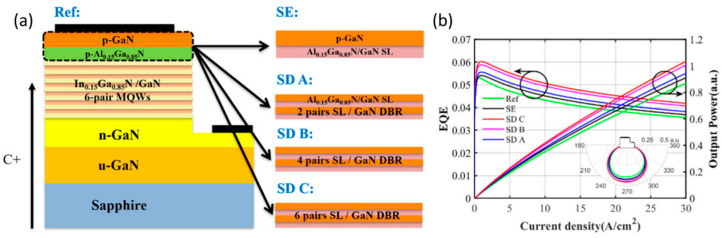
(**a**) Schematic diagram of µLEDs. (**b**) The output power and EQE as a function of current density. Reproduced from Ref. [110], with the permission of Optical Society of America.

In addition, a numerical investigation is numerically done on the function of a superlattice distributed Bragg reflector (SL DBR) as the p-type electron blocking layer in the GaN-based µLED [110]. The 2, 4, and 6 pairs of DBRs in LED devices are represented by SD A, SD B, and SD C in Figure 13a, respectively. Figure 13b shows that the EQE of SD C is 18.5% greater than that of Ref, and LEE enhances with the number of DBsR pairs.

An inclined mesa favors the optical reflection towards the substrate and can be an easier approach to increasing the LEE [111,112,113]. Hang et al. have numerically investigated and demonstrated the impact of different inclination mesa angles on the optical and electrical properties of GaN-based µLEDs [113]. The simulation study has shown that enhanced LEE can be obtained by appropriately reducing the mesa angle of µLEDs. However, there is a trade-off between LEE and IQE in that when the inclined mesa angle is reduced, despite the enhanced LEE, the increased electric field in the sidewall region enhances the QCSE therein, and the non-radiative recombination rate induced by surface defects will increase. Therefore, the inclined mesa angle should be carefully designed and optimized. Due to the excellent scattering ability of the tilted countertop sidewall of µLEDs, and the certain thickness of the air layer between the n-GaN layer and the sapphire substrate, this can be utilized. Jia et al. propose the air-cavity patterned sapphire substrate (AC-PSS) as the light filter [114]. By combining the filtering effect of AC-PSS and the scattering effect of the tilted countertop sidewall, LEE can be enhanced while also improving optical crosstalk. Although manufacturing the above air layer is challenging in reality, its structure provides an important reference value for enhancing LEE.

## 5. Conclusions

In summary, we have reviewed the factors that affect the performance of µLEDs as chip size decreases. Surface recombination and sidewall damage have more detrimental effects on the EQE of µLEDs because of their high perimeter-to-area ratio. In order to improve the reliability of the device, on the one hand, the combination of chemical treatment and ALD sidewall passivation, as well as optimization of thermal annealing time or direct epitaxial growth method, are used to effectively suppress non-ideal characteristics caused by device manufacturing. Dislocation density can be decreased by growing µLEDs on nanopatterned substrates and buffer insertion layers. One may use tunnel junctions instead of ITO to adjust the current extension length and maintain a distance between the current and the edge of the table to effectively suppress the surface recombination rate. To suppress QCSE, several methods of epitaxial structure optimization have been adopted, such as epitaxial growth on non-polar or semi-polar planes, strain release from nano-LED structures, and the design of suitably thin quantum barriers. In addition, we also reviewed some approaches to further optimizing the EQE by increasing the LEE, such as ODR, the SL DBR, and so on. We believe that there are many ways to optimize the EQE of µLEDs to be explored, inspiring researchers to make high-brightness µLEDs.

## Figures and Tables

**Table 1:** Comparison of LCD, OLED, and µLED [8].

Features	LCD	OLED	µLED
view angle	max. 89°	max. 89°	max. 180°
display type	backlit	self-emissive	self-emissive
pixel size	large (min 32 μm)	medium (min 18 μm)	small (submicrometer)
power efficiency	medium	Medium	high
response time	ms	Μs	ns
PPI	max. 30,000 PPI	max. 1433 PPI	max. 30,000 PPI
temperature stability	−20 to 80 °C	−50 to 70 °C	−100 to 120 °C
contrast ratio	5000:1	>10,000:1	>1,000,000:1
service lifetime	30,000−60,000 h	10,000 h	>100,000 h
cost	low	Medium	high

**Table 2:** Optoelectronic performances at the 83 A/cm^2^ current density with and without EBI-based μLEDs. Reproduced from Ref. [108], with the permission of Elsevier.

µ-LEDs Type	EQE (%)	LEE (%)	IQE (%)	LOP (mW)
With EBI	37.2	81.2	54.5	9.44
Without EBI	32.6	70.6	53.1	8.27
